# Designing Antiferromagnetic Spin-1/2 Chains in Janus Fullerene Nanoribbons

**DOI:** 10.1021/acs.nanolett.5c06318

**Published:** 2026-05-01

**Authors:** Bo Peng, Michele Pizzochero

**Affiliations:** † Theory of Condensed Matter Group, Cavendish Laboratory, 2152 University of Cambridge, Cambridge CB3 0HE, United Kingdom; ‡ Department of Physics, 1555 University of Bath, Bath BA2 7AY, United Kingdom; ¶ School of Engineering and Applied Sciences, Harvard University, Cambridge, Massachusetts 02138, United States

**Keywords:** spin chain, nanoribbons, monolayer fullerene networks, first-principles

## Abstract

We designed antiferromagnetic spin-1/2 chains in fullerene nanoribbons by introducing extra C_60_ cages at one of their edges. The resulting odd number of intermolecular bonds induces an unpaired π-electron and hence a quantized magnetic moment in otherwise nonmagnetic nanoribbons. We further reveal the formation of an antiferromagnetic ground state upon the linear arrangement of spin-1/2 C_60_ cages that is insensitive to the specific structural motifs. Janus fullerene nanoribbons may offer an experimentally accessible route to magnetic edge states in low-dimensional carbon nanostructures, possibly serving as a versatile nanoarchitecture for scalable spin-based devices and the exploration of many-body quantum phases.

## Graphical abstract

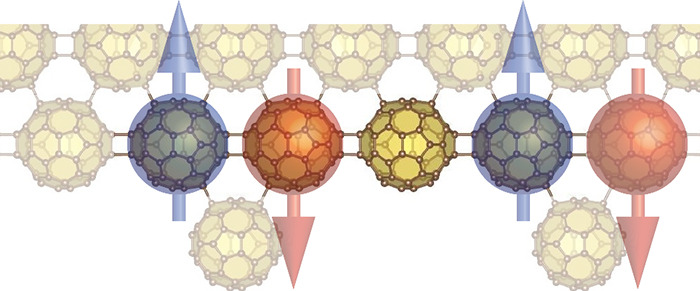

Spin-1/2 chains are prototypical many-body systems featuring an inherently quantum mechanical spin degree of freedom where quantum criticality can be continuously tuned via the application of a magnetic field.^1,2^ Theoretical models, most notably the Heisenberg antiferromagnetic chain, have long predicted exotic quantum properties.^3–6^ Yet, only recent experimental advances have enabled their direct observation,^7–9^ further motivating the search of one-dimensional materials as a platform to explore quantum magnetism and correlated phenomena. In this vein, nanoribbons of graphene hosting π-electron magnetism^10,11^ have emerged as promising candidates, especially in light of their potential applications ranging from electronics,^12^ spintronics,^13,14^ or qubits,^15–18^ to the realization of topological^19–23^ and Majorana states.^24^ Nevertheless, achieving atomically precise, chemically stable graphene nanoribbons with well-defined magnetic edges remains a significant challenge.

The recent synthesis of monolayer networks of C_60_ ^25^ provides new opportunities for designing nanoribbons with tunable edge geometries upon confinement in one dimension.^26,27^ After our predictions on experimental feasibility of fullerene networks to form wide nanoribbons,^26^ the synthesis of wide fullerene nanoribbons has been reported.^28^ Additionally, rich structural phases have been found in 2D fullerene networks, as predicted computationally^29–38^ and verified experimentally,^39–41^ where various nanoribbons can be envisioned. While the Kekulé valence structure of the C_60_ molecule has been well understood,^42–44^ the possibility to realize π-electron magnetism in their extended networks by creating unpaired electrons remains unexplored. This approach could provide valuable insights into designing C_60_ nanoribbon-based spin chains in the context of quantum magnetism.

Here, we use first-principles calculations to establish general design principles to achieve tailored magnetism in otherwise nonmagnetic fullerene nanoribbons. This is accomplished by the introduction of extra C_60_ cages at one of the edges, leading to the formation of Janus fullerene nanoribbons. As a result of the odd number of intermolecular bonds induced by the extra cage, one unpaired electron is created and confined within the C_60_ molecule, acting as a spin-1/2 center. Neighboring C_60_ units at the edges couple antiferromagnetically, effectively realizing a quantum spin chain embedded in a fullerene nanoribbon. The magnetic edge states are robust for different spatial arrangement of the extra cages along the edges. Overall, our findings enable the rational design and engineering of magnetic fullerene nanoribbons through a precise functionalization of their edges.

To introduce magnetic edge states, we added an extra C_60_ cage at one of the edges of the nanoribbon. This leads to the Janus fullerene nanoribbon in Figure 1(a). As shown in Figure 1(a), the C_60_ cages denoted in green circles near the extra fullerene cage have an odd number of intermolecular bonds and hence host an unpaired electron, leading to π-electron magnetism.

**Figure 1:**
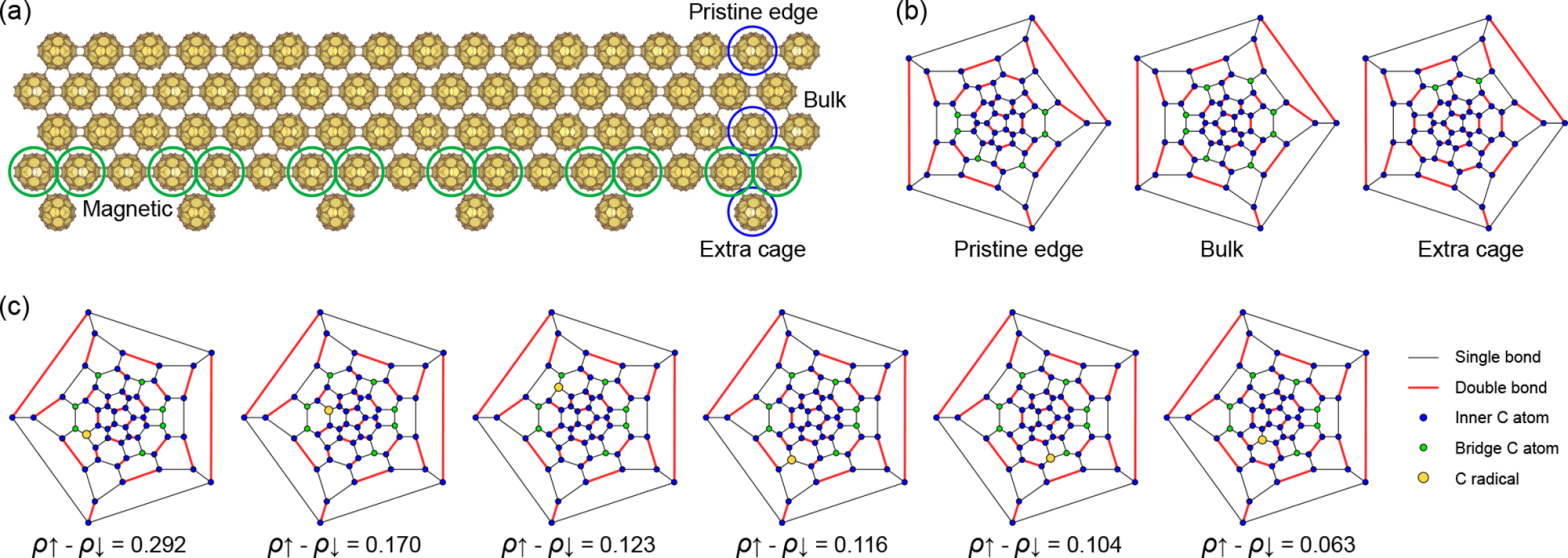
(a) Crystal structure of a Janus fullerene nanoribbon, along with the corresponding Schlegel diagrams for (b) nonmagnetic and (c) magnetic C_60_ cages.

To better understand the origin of π-electron magnetism in Janus fullerene nanoribbons, it is instructive to first consider nonmagnetic, pristine fullerene nanoribbons and their constituent fullerene units. For isolated C_60_ cages, the resonance structure features 30 double bonds and 60 single bonds and hence has no magnetism. At the pristine edge of the nanoribbon displayed in Figure 1(a), there are two intermolecular [2 + 2] cycloaddition bonds along the nanoribbon length and two intermolecular C–C single bonds across the nanoribbon width. The Schlegel diagram depicted in Figure 1(b) shows one resonance structure of the fullerene at the pristine edge. The six carbon atoms forming intermolecular *sp* ^3^ bonds are colored green, which are fully saturated. For the other carbon atoms indicated in blue, the *sp* ^2^ hybridization results in three single σ bonds (the black line), while two neighboring π electrons join one of the three single bonds to form a double bond (the red line). For the C_60_ cages on the pristine edge, each unsaturated carbon atom in blue is surrounded by two single bonds and one double bond in Figure 1(b), leaving no unpaired electron and hence no magnetic moment in the entire C_60_ cage. Similarly, the fullerene cage in the bulk has no unpaired electron and is nonmagnetic, as well as the extra fullerene cage, which is due to the even number of intermolecular bonds, as shown in Figure 1(b).

For C_60_ cages in the green circles of Figure 1(a), however, there are two intermolecular [2 + 2] cycloaddition bonds and three intermolecular C–C single bonds, leaving one unpaired electron. The unpaired electron in each magnetic C_60_ cage with an odd number of interfullerene bonds has fully quantized spin-1/2, which is confined within the fullerene unit but delocalized at six main carbon sites due to the resonance structure. Figure 1(c) shows the Schlegel diagram of the six magnetic carbon atoms in their resonance structure, confirming the quasi-localized nature of the unpaired electron (the yellow point). The Mulliken population difference between spin up and spin down (ρ_↑_–ρ_↓_) shows that these six carbon atoms contribute to more than 86.8% of the total magnetic moment within the buckyball, i.e., 1 μ_B_ per magnetic C_60_.

We next examined the magnetic order of the Janus fullerene nanoribbons. In Figure 2(a), we compare the difference in total energy between the nonmagnetic, ferromagnetic, and antiferromagnetic phases, together with their corresponding spin orientations. The ferromagnetic phase is energetically more favorable by 103.2 meV (60.0 meV) per unit cell than the nonmagnetic phase, while the antiferromagnetic nanoribbon is 9.0 meV (12.0 meV) per unit cell lower in energy than the ferromagnetic phase from the PBE (PBEsol) functional. We thus conclude that the antiferromagnetic phase is the most thermodynamically stable phase in the absence of external fields.

**Figure 2:**
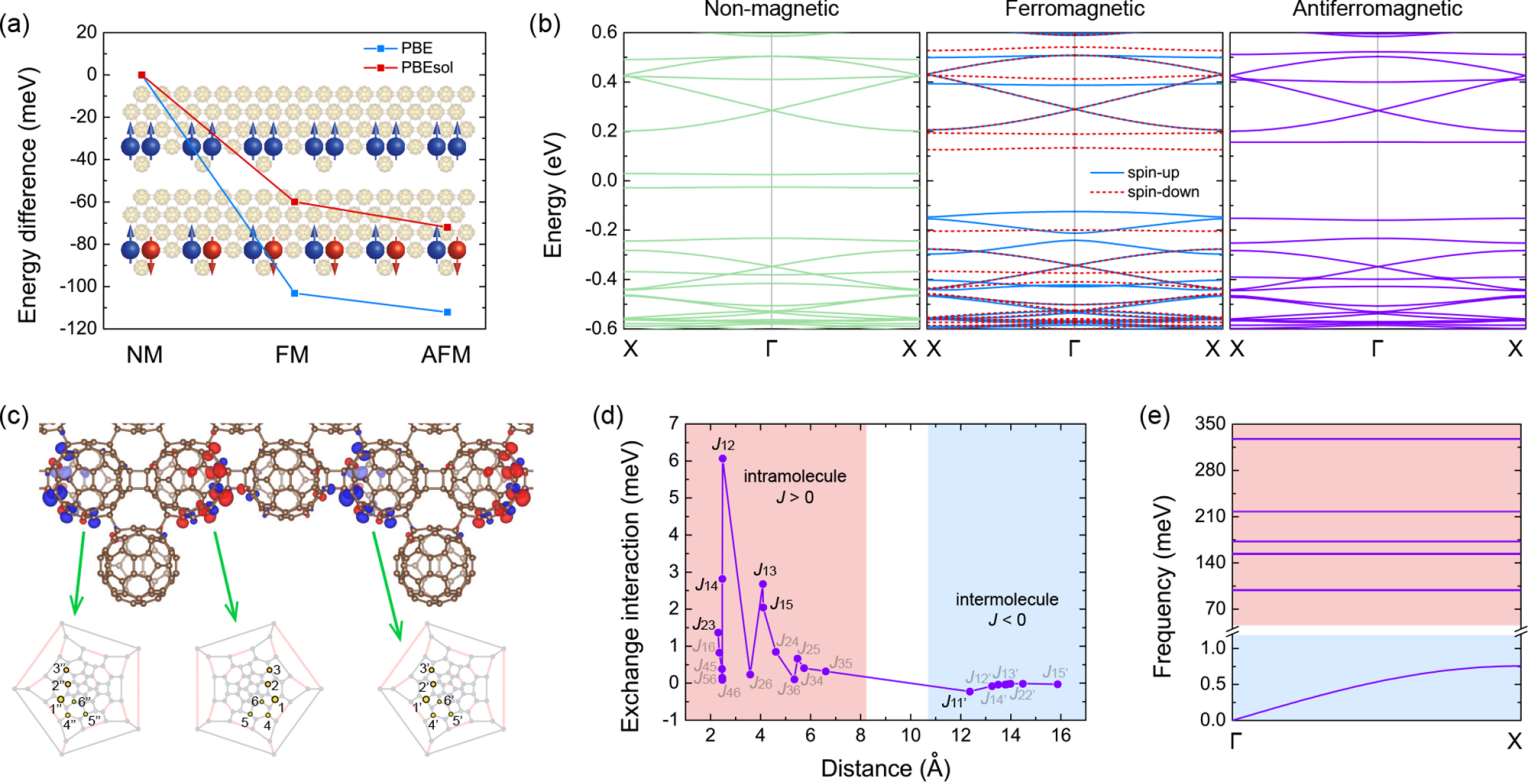
(a) Energy difference and (b) electronic band structure of Janus fullerene nanoribbons in the nonmagnetic, ferromagnetic, and antiferromagnetic state. (c) Spin densities and Schlegel diagrams for antiferromagnetic fullerene chains. The blue and red color represent spin-up and spin-down states, respectively. (d) Magnetic exchange interaction between magnetic carbon sites as a function of their distance and (e) magnon spectrum.

We gain insight into the electronic properties of the three magnetic phases of Janus fullerene nanoribbons by studying their band structures, as shown in Figure 2(b). The nonmagnetic phase has two flat edge states as the highest valence band and lowest conduction band, respectively, with a band gap of 0.09 eV. In the ferromagnetic phase, while most of the bands remain doubly degenerate, the two top valence bands are spin-up states from the two magnetic fullerene cages, while the two bottom conduction bands are spin-down states from the same two C_60_ cages. These bands are mainly contributed by the fullerene units in green circles of Figure 1(a), as confirmed by the projected density of states in Figure S1 in the Supporting Information. There are also several degenerate bands split into one spin-up band and one spin-down band around −0.25, 0.4, and 0.5 eV, respectively. For the stable antiferromagnetic phase, the deep valence bands and higher conduction bands remain the same as the nonmagnetic phase, whereas the highest valence band and lowest conduction band exhibit a larger splitting with a band gap of 0.31 eV.

We then examine the isotropic exchange interactions between magnetic carbon atoms as a function of the distance between these carbon sites. Figure 2(c) shows the spin densities of the antiferromagnetic fullerene chains. We label the magnetic sites in the second C_60_ cages (from left to right) in Figure 2(c) as 1–6 from the largest to smallest magnetic moment. The exchange interactions between magnetic atoms are computed from the Green’s function^45,46^ as implemented in the TB2J package.^47^ Within a C_60_ cage (<8 Å), all intramolecular exchange interactions are ferromagnetic, as shown in Figure 2(d). Only *J*_23_, *J*_14_, *J*_12_, *J*_13_, and *J*_15_ have relatively larger exchange interaction (>1 meV), especially *J*_12_ = 6.065 meV. On the other hand, all intermolecular interactions are antiferromagnetic. Interestingly, the interfullerene interactions between the nearest neighboring fullerene units, i.e., between 1–6 and 1″–6″in Figure 2(c), are all zero. However, the second nearest neighboring C_60_ cages, i.e., 1–6 and 1′–6′ in Figure 2(c), have much stronger interfullerene exchange interactions, with the strongest one being *J*_11′_ = −0.222 meV while the rest |*J*_*ii*_′| < 0.1 meV. Beyond 17 Å, the exchange interactions vanish. These results indicate weak antiferromagnetic interactions in the spin-1/2 chain.

Using the calculated isotropic exchange terms, we obtain the magnon dispersion of the stable antiferromagnetic phase under the Holstein–Primakoff transformation^48^ by diagonalizing the bosonic Hamiltonian.^49^ As shown in Figure 2(e), the low-frequency magnons below 1 meV show linear dispersion around the Γ point, indicating typical antiferromagnetic features.^50^ These low-frequency magnons are doubly degenerate, corresponding to the two carbon cages with opposite spins. The magnon dispersion has non-negative values with a global minimum at Γ, demonstrating that the antiferromagnetic order is indeed the magnetic ground state,^51^ in line with our energetic analysis. It should be noted that TB2J underestimates the exchange interaction strength sometimes by 1 order of magnitude comparing to the four-state method.^52,53^ The total energy difference between the ferromagnetic and antiferromagnetic phases is around 10 meV, while the intermolecular coupling from TB2J is much smaller, likely due to the localized atomic basis set employed in the SIESTA package.^54–56^ Nevertheless, this implies the plausibility that the antiferromagnetic behaviors in Janus fullerene nanoribbons tend to be even stronger than the TB2J-based predictions. Despite that, the energy difference between the ferromagnetic and antiferromagnetic phases is rather small (9–12 meV). It is therefore expected that the magnetic order is quite fragile against thermal fluctuations for temperatures beyond 100–150 K, depending on effects such as thermal expansion,^35^ electron–phonon coupling,^57,58^ and spin–phonon interactions.

Finally, we study the magnetic edge states for various spatial arrangements of extra C_60_ cages at the edges. We focus on the ferromagnetic phase because of the spin-polarized nature of its magnetic edge states, which is particularly appealing for spintronics. We first increase the spacing between the extra C_60_ cages located at the same edge, as shown in Figure 3(a). The cohesive energy *E*_C_ is defined as

1 $$E_{C}=E_{\mathrm{nanoribbon}}/n-E_{C_{60}}$$

where *E*_nanoribbon_ is the total energy of the Janus nanoribbon, *n* is the number of C_60_ cages in the nanoribbon, and *E*_C_60__ is the total energy of one isolated C_60_ cage. The increased spacing between the extra C_60_ cages leads to a cohesive energy *E*_C_ lowering of 4.0 and 6.4 meV per C_60_ unit respectively in the presence and absence of the 3*s* and 3*p* diffuse orbitals (single-ζ with fixed radii at 10 Bohr). This agrees with our previous results showing enhanced thermodynamic stability of fullerene nanoribbons with increased number of fullerene cages in the unit cell.^26^ Interestingly, nanoribbons with larger spacing between the extra C_60_ cages exhibit distinct band structures for the ferromagnetic phase displayed in Figure 3(b) but the same spin-1/2 behavior.

**Figure 3:**
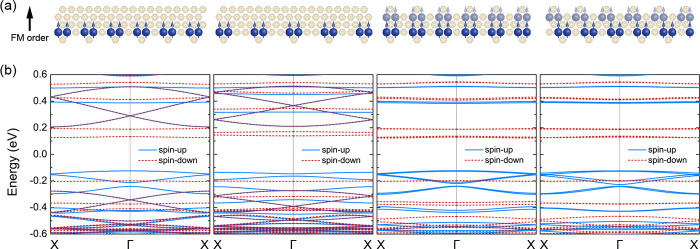
(a) Crystal structure of fullerene nanoribbons featuring various arrangements of extra C_60_ cages at the edges and (b) their electronic band structure in the ferromagnetic phase.

Similarly, we can create chevron-like fullerene nanoribbons^59^ where both edges host π-electron magnetism, doubling the magnetic moment per unit cell compared to Janus fullerene nanoribbons. For these chevron-like nanoribbons, two configurations are possible, depending on whether the two extra C_60_ units on the opposite edges are aligned (leading to a space group of *P*2/*m*, No. 10) or misaligned (space group *P*1̅, No. 2) across the nanoribbons. Despite the different space groups, however, the two ferromagnetic chevron nanoribbons have nearly identical band structures with a tiny total energy difference within 3.4 meV.

The experimental realization of fullerene nanoribbons is promising due to recent advances in the synthesis of monolayer fullerene networks.^25^ Most recently, shortly after our predictions on experimental feasibility in realizing wide fullerene nanoribbons in the quasi-tetragonal phase,^26^ it has been reported that rectangular faceted 2D sheets of covalently linked C_60_ in this specific phase can be formed under continuous flow with uniform dimensions of 20 μm in length and 6 μm in width.^28^ Apart from the large width, the maximum length of the C_60_ rectangular sheet is up to 46 μm, much longer than that of graphene nanoribbons (up to 0.05–10 μm,^60,61^ as far as we know), suggesting the inherent ability for fullerene molecules to be self-assembled into large, ordered structures. The bottom-up arrangement of C_60_ molecules with a scanning tunneling microscope tip has been realized for three decades,^62,63^ providing feasible routes to engineer the edge motifs that host π-electron magnetism.

Our findings envision Janus fullerene nanoribbons as a versatile platform to design quantum spin chains for a wide range of applications from qubit entanglement to spintronics. Comparing to phosphorene nanoribbons with magnetic edge states,^64–68^ carbon has intrinsically weaker spin–orbit coupling and negligible hyperfine interaction due to the low isotope ratio of ^13^C, ∼1.1%,^14,18^ which is in direct contrast to ^31^P of 100% isotope abundance with nuclear spin *I* = 1/2 and large hyperfine constants.^69,70^ Therefore, the spin coherence time in fullerene nanoribbons is expected to be much longer, making the proposed Janus fullerene nanoribbons promising building blocks for scalable qubit systems. Compared to graphene nanoribbons, individual spin-1/2 C_60_ cages can serve as quantum dots with localized spins and controllable exchange interactions. In addition, quantum spin chains realized by Janus fullerene nanoribbons can enable scalable architectures for designing quantum simulators, tunable spintronic devices for exploiting spin-polarized currents, and versatile platforms for realizing emergent topological phases and Majorana modes when coupled with superconductors. The same building blocks can also be extended beyond quasi-1D nanoribbons for realizing even more complex condensed matter systems in 2D,^71^ such as quantum anomalous Hall insulators^72^ and altermagnetic Shastry–Sutherland lattices that can be continuously tuned into a quantum spin liquid phase.^73^ Additionally, the fullerene-based nanoribbons are chemically stable, offering scalable platforms that have the potential to operate at room temperature. Overall, the combination of quantized spin, structural flexibility, and long spin coherence places Janus fullerene nanoribbons at the forefront of candidates for next-generation carbon-based quantum technologies.

In summary, our work establishes a strategy to create metal-free, magnetic 1D spin chains based on Janus fullerene nanoribbons. By introducing extra C_60_ cages to pristine edges, we demonstrate that π-electron magnetism can arise as a result of the odd number of intermolecular bonds, with quantized magnetic moments residing primarily at the two neighboring C_60_ cages on the edge. This gives rise to an antiferromagnetic *S* = 1/2 chain with weak intermolecular exchange interactions. Spin-1/2 fullerene nanoribbons offer high chemical stability and feasible structural control, lowering the barrier for experimental realization. Beyond their fundamental importance for probing quantum magnetism, these nanoribbons present promising opportunities for quantum technologies such as spintronic devices and qubit systems by virtue of the weak spin–orbit and hyperfine interactions intrinsic to carbon, which ensures long spin coherence times. Hence, the tunability and scalability of these fullerene-based platforms may pave the way toward integrating carbon-based magnetic architectures into next-generation quantum information systems.

## Supplementary Material



## Notes

Computational details; projected density of states (PDF)
