# Supplementary material for: Designing Antiferromagnetic Spin-1/2 Chains in Janus Fullerene Nanoribbons
Source: Nano Lett. 2026 May 1;26(18):6034–9. doi: 10.1021/acs.nanolett.5c06318 (PMC13178130; doi:10.1021/acs.nanolett.5c06318)
Supplement: Supplementary file 1 [file nl5c06318_si_001.pdf]

Supporting Information for "Designing Antiferromagnetic Spin-1/2 Chains in Janus Fullerene Nanoribbons"

Bo Peng[*,†] and Michele Pizzochero[*,‡,¶]

† *Theory of Condensed Matter Group, Cavendish Laboratory, University of Cambridge, Cambridge CB3 0HE, United Kingdom*

‡ *Department of Physics, University of Bath, Bath BA2 7AY, United Kingdom*

¶ *School of Engineering and Applied Sciences, Harvard University, Cambridge, Massachusetts 02138, United States*

E-mail: bp432@cam.ac.uk; mp2834@bath.ac.uk

Computational details

Our computational models of fullerene nanoribbons contain 660–1020 carbon atoms in the unit cell. Regarding the choices of the width for the nanoribbons, we have shown that the band width is converged within 10 meV when the nanoribbon widths $W > 4$ (i.e., > 30 Å).[1] For Janus fullerene nanoribbons, the band edges are dominated by the green fullerene cages on the Janus edge, which exhibit flat edge bands with strong localised features, as confirmed by the projected density of states in Fig. S1. Therefore, the electronic properties of the green cages remain localised with increased nanoribbon width W. Similarly, the exchange interactions are relatively short-ranged and decay into zero beyond 20 Å, as shown in Fig. 2(d) in the main text. This justifies our choice of the nanoribbon widths.

Density functional theory (DFT) calculations [2,3] are performed under the spin-polarised, generalised-gradient approximation (GGA) of Perdew, Burke, and Ernzerhof (PBE),[4] as implemented in the SIESTA package.[5–7] A double-ζ plus polarisation (DZP) basis set is used with an energy cutoff of 400 Ry and a reciprocal space sampling of 10 k-points along the periodic direction for structural relaxation. A vacuum spacing in the non-periodic directions larger than 20 Å is adopted throughout all the calculations. Both the lattice constant and atomic positions are fully relaxed using the conjugate gradient method [8] with a tolerance on forces of 0.02 eV/Å.

The total energy of the Janus nanoribbon is 295 meV lower than the combined energy of the pristine nanoribbon and an isolated C_{60}. However, the edge-functionalisation of fullerene nanoribbons is no longer energetically favourable after including both $3s$ and $3p$ diffuse orbitals (single-ζ) with fixed radii at 10 Bohr. However, the inclusion of diffuse orbitals only changes the relative energy difference between the ferromagnetic and antiferromagnetic phases of Janus fullerene nanoribbons by less than 1 meV. We also compute the total energies of the non-magnetic, ferromagnetic, and antiferromagnetic phases using the PBEsol exchange-correlation functional [9] from the PseudoDojo database,[10] which predicts a similar trend as shown in Fig. 2(a) in the main text.

For band structure calculations, the k-points are increased to 100 to sample the high-symmetry line. For monolayer qHP networks, the inclusion of the Grimme's D3 dispersion corrections [11] leads to a decrease in lattice constants by merely 0.3%.[12,13] We therefore neglect the van der Waals interactions hereafter.

The Mulliken population analysis between spin up ($\rho_\uparrow$) and down ($\rho_\downarrow$) is applied to study the magnetic moment at each carbon atom. We choose magnetic atoms with $|\rho_\uparrow - \rho_\downarrow| > 0.06$, which contribute to nearly 90% of the total magnetic moment. The spin densities in Fig. 2(c) are computed using the C2X code [14] and then visualised in VESTA.[15] The exchange interactions between magnetic atoms are computed from the Green's function [16,17] based on the Wannier tight-binding Hamiltonian,[18–23] as implemented in the TB2J package.[24] Such in-

teractions are found to vanish beyond 17 Å. For antiferromagnetic nanoribbons, the magnons are diagonalised based on the bosonic Hamiltonian [25] under the Holstein-Primakoff transformation,[26] as implemented in the MAGNOPY package that has been widely employed to study low-dimensional antiferromagnets.[27,28]

Projected density of states

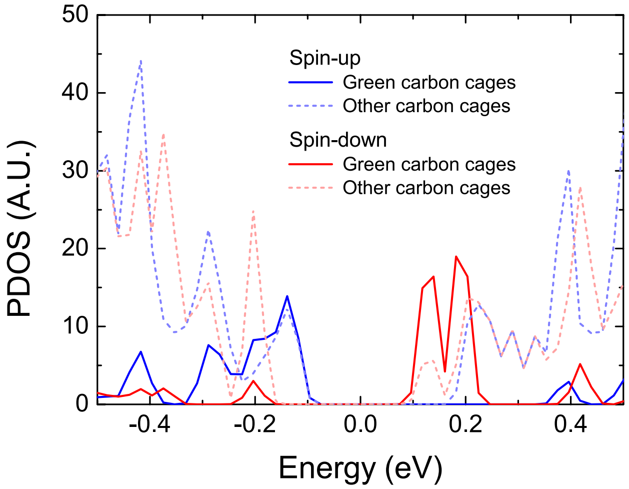

Figure S1: Projected density of states around the Fermi energy for ferromagnetic fullerene nanoribbons, where spin-up (blue) and spin-down (red) states are plotted for both the green carbon cages (solid line) and the other carbon cages (short dashed line).

References

(1) Peng, B.; Pizzochero, M. Electronic Structure of Fullerene Nanoribbons. ACS Nano **2025**, 19, 29637–29645.

(2) Hohenberg, P.; Kohn, W. Inhomogeneous Electron Gas. Phys. Rev. **1964**, 136, B864–B871.

(3) Kohn, W.; Sham, L. J. Self-Consistent Equations Including Exchange and Correlation Effects. Phys. Rev. **1965**, 140, A1133–A1138.

(4) Perdew, J. P.; Burke, K.; Ernzerhof, M. Generalized Gradient Approximation Made Simple. Phys. Rev. Lett. **1996**, 77, 3865–3868.

(5) Soler, J. M.; Artacho, E.; Gale, J. D.; García, A.; Junquera, J.; Ordejón, P.; Sánchez-Portal, D. The SIESTA method for ab initio order-N materials simulation. J. Phys.: Condens. Matter **2002**, 14, 2745–2779.

(6) Artacho, E.; Anglada, E.; Diéguez, O.; Gale, J. D.; García, A.; Junquera, J.; Martin, R. M.; Ordejón, P.; Pruneda, J. M.; Sánchez-Portal, D.; Soler, J. M. The SIESTA method; developments and applicability. J. Phys.: Condens. Matter **2008**, 20, 064208.

(7) García, A. et al. Siesta: Recent developments and applications. J. Chem. Phys. **2020**, 152, 204108.

(8) Payne, M. C.; Teter, M. P.; Allan, D. C.; Arias, T. A.; Joannopoulos, J. D. Iterative minimization techniques for *ab initio* total-energy calculations: molecular dynamics and conjugate gradients. Rev. Mod. Phys. **1992**, 64, 1045–1097.

(9) Perdew, J. P.; Ruzsinszky, A.; Csonka, G. I.; Vydrov, O. A.; Scuseria, G. E.; Constantin, L. A.; Zhou, X.; Burke, K. Restoring the Density-Gradient Expansion for Exchange in Solids and Surfaces. Phys. Rev. Lett. **2008**, 100, 136406.

(10) van Setten, M.; Giantomassi, M.; Bousquet, E.; Verstraete, M.; Hamann, D.; Gonze, X.; Rignanese, G.-M. The PseudoDojo: Training and grading a 85 element optimized norm-conserving pseudopotential table. Computer Physics Communications **2018**, 226, 39–54.

(11) Grimme, S.; Antony, J.; Ehrlich, S.; Krieg, H. A consistent and accurate ab initio parametrization of density functional dispersion correction (DFT-D) for the 94 elements H-Pu. J. Chem. Phys. **2010**, 132, 154104–.

(12) Peng, B. Monolayer Fullerene Networks as Photocatalysts for Overall Water Splitting. J. Am. Chem. Soc. **2022**, 144, 19921–19931.

(13) Shearsby, D.; Wu, J.; Yang, D.; Peng, B. Tuning electronic and optical properties of 2D polymeric C60 by stacking two layers. Nanoscale **2025**, 17, 2616–2620.

(14) Rutter, M. J. C2x: A tool for visualisation and input preparation for Castep and other electronic structure codes. Computer Physics Communications **2018**, 225, 174–179.

(15) Momma, K.; Izumi, F. VESTA 3 for three-dimensional visualization of crystal, volumetric and morphology data. Journal of Applied Crystallography **2011**, 44, 1272–1276.

(16) Liechtenstein, A.; Katsnelson, M.; Antropov, V.; Gubanov, V. Local spin density functional approach to the theory of exchange interactions in ferromagnetic metals and alloys. Journal of Magnetism and Magnetic Materials **1987**, 67, 65–74.

(17) Korotin, D. M.; Mazurenko, V. V.; Anisimov, V. I.; Streltsov, S. V. Calculation of exchange constants of the Heisenberg model in plane-wave-based methods using the Green's function approach. Phys. Rev. B **2015**, 91, 224405.

(18) Marzari, N.; Vanderbilt, D. Maximally localized generalized Wannier functions for composite energy bands. Phys. Rev. B **1997**, 56, 12847–12865.

(19) Souza, I.; Marzari, N.; Vanderbilt, D. Maximally localized Wannier functions for entangled energy bands. Phys. Rev. B **2001**, 65, 035109.

(20) Mostofi, A. A.; Yates, J. R.; Lee, Y.-S.; Souza, I.; Vanderbilt, D.; Marzari, N. Wannier90: A tool for obtaining maximally-localised Wannier functions. Computer Physics Communications **2008**, 178, 685–699.

(21) Marzari, N.; Mostofi, A. A.; Yates, J. R.; Souza, I.; Vanderbilt, D. Maximally localized Wannier functions: Theory and applications. Rev. Mod. Phys. **2012**, 84, 1419–1475.

(22) Mostofi, A. A.; Yates, J. R.; Pizzi, G.; Lee, Y.-S.; Souza, I.; Vanderbilt, D.; Marzari, N. An updated version of Wannier90: A tool for obtaining maximally-localised Wannier functions. Computer Physics Communications **2014**, 185, 2309–2310.

(23) Pizzi, G. et al. Wannier90 as a community code: new features and applications. J. Phys.: Condens. Matter **2020**, 32, 165902.

(24) He, X.; Helbig, N.; Verstraete, M. J.; Bousquet, E. TB2J: A python package for computing magnetic interaction parameters. Computer Physics Communications **2021**, 264, 107938.

(25) Colpa, J. Diagonalization of the quadratic boson hamiltonian. Physica A: Statistical Mechanics and its Applications **1978**, 93, 327–353.

(26) Holstein, T.; Primakoff, H. Field Dependence of the Intrinsic Domain Magnetization of a Ferromagnet. Phys. Rev. **1940**, 58, 1098–1113.

(27) Rybakov, A.; Boix-Constant, C.; Alba Venero, D.; van der Zant, H. S. J.; Mañas-Valero, S.; Coronado, E. Probing Short-Range Correlations in the van der Waals Magnet CrSBr by Small-Angle Neutron Scattering. Small Sci. **2024**, 4, 2400244–.

(28) Boix-Constant, C.; Rybakov, A.; Miranda-Pérez, C.; Martínez-Carracedo, G.; Ferrer, J.; Mañas-Valero, S.; Coronado, E. Programmable Magnetic Hysteresis in Orthogonally-Twisted 2D CrSBr Magnets via Stacking Engineering. Adv. Mater. **2025**, 37, 2415774–.
